# Zika Virus-Induced Neuronal Apoptosis via Increased Mitochondrial Fragmentation

**DOI:** 10.3389/fmicb.2020.598203

**Published:** 2020-12-23

**Authors:** Shu Yang, Kirill Gorshkov, Emily M. Lee, Miao Xu, Yu-Shan Cheng, Nuo Sun, Ferri Soheilian, Natalia de Val, Guoli Ming, Hongjun Song, Hengli Tang, Wei Zheng

**Affiliations:** ^1^National Center for Advancing Translational Sciences, National Institutes of Health, Bethesda, MD, United States; ^2^Department of Physiology and Cell Biology, The Ohio State University Wexner Medical Center, Columbus, OH, United States; ^3^Electron Microscopy Laboratory, National Cancer Institute, Center for Cancer Research, Leidos Biomedical Research, Frederick National Laboratory, Frederick, MD, United States; ^4^Department of Neuroscience, Mahoney Institute for Neurosciences, University of Pennsylvania, Philadelphia, PA, United States; ^5^Department of Biological Science, Florida State University, Tallahassee, FL, United States

**Keywords:** Zika virus, mitochondrial fragmentation, MFN2, apoptosis, mitofusin

## Abstract

The 2015 to 2016 outbreak of Zika virus (ZIKV) infections in the Americas coincided with a dramatic increase in neurodevelopmental abnormalities, including fetal microcephaly, in newborns born to infected women. In this study, we observed mitochondrial fragmentation and disrupted mitochondrial membrane potential after 24 h of ZIKV infection in human neural stem cells and the SNB-19 glioblastoma cell line. The severity of these changes correlated with the amount of ZIKV proteins expressed in infected cells. ZIKV infection also decreased the levels of mitofusin 2, which modulates mitochondria fusion. Mitochondrial division inhibitor 1 (Mdivi-1), a small molecule inhibiting mitochondria fission, ameliorated mitochondria disruptions and reduced cell death in ZIKV-infected cells. Collectively, this study suggests that abnormal mitochondrial fragmentation contributes to ZIKV-induced neuronal cell death; rebalancing mitochondrial dynamics of fission-fusion could be a therapeutic strategy for drug development to treat ZIKV-mediated neuronal apoptosis.

## Introduction

Zika virus (ZIKV) belongs to the *Flavivirus* genus in the *Flaviviridae* family of RNA viruses (Ming et al., 2016). Similar to other flaviviruses, ZIKV is most commonly transmitted through the bites of infected *Aedes* mosquitoes. However, unlike other flaviviruses, ZIKV can also be sexually and vertically transmitted (Musso et al., 2015; Venturi et al., 2016; Mesci et al., 2018). The single-strand, positive-sense RNA genome in ZIKV encodes a large polyprotein. It is then post-translationally cleaved by both viral and host proteases to form many proteins. The three structural proteins are the capsid (C), the precursor membrane (prM) that is further cleaved in the maturing virion to form the membrane (M) protein, and the envelope (ENV) (Ye et al., 2016). Further cleavages result in seven non-structural proteins: NS1, NS2A, NS2B, NS3, NS4A, NS4B, and NS5 (Garcia-Blanco et al., 2016). The structural proteins, together with the single-strand RNA genome, form the enveloped ZIKV spherical particle with a diameter of approximately 40 nm (Chambers et al., 1990). Although 80% of ZIKV-infected individuals are asymptomatic or exhibit mild symptoms that include fever, malaise, rash, and conjunctivitis (Ioos et al., 2014; Wang et al., 2016), mounting evidence has linked the ZIKV outbreak in the Americas to a marked increase of newborn microcephaly, a developmental abnormality resulting in diminished head size and brain formation (De Carvalho et al., 2016; Fauci and Morens, 2016; Hazin et al., 2016; Oliveira Melo et al., 2016; Schuler-Faccini et al., 2016). Several case reports have shown the presence of ZIKV particles in the microcephalic fetal brain (Driggers et al., 2016; Mecharles et al., 2016; Mlakar et al., 2016). ZIKV infects fetal brain tissue directly and prominently affects neural stem and progenitor cells, leading to abnormal cell differentiation, decreased proliferation, and cell death (Garcez et al., 2016; Li et al., 2016; Ming et al., 2016; Qian et al., 2016; Tang et al., 2016; Gabriel et al., 2017). However, the specific molecular mechanism of ZIKV infection-induced fetal microcephaly remains unclear.

Mitochondria are double-membrane-bound organelles with a wide range of cellular functions, including ATP generation, programmed cell death, and calcium homeostasis, as well as the biosynthesis of amino acids, lipids, nucleotides, and heme (West et al., 2011). Mitochondria are dynamic organelles that constantly undergo fusion (two or more independent mitochondria fusing into a single organelle) and fission (the opposing reaction of fusion that splits one organelle into two or more structures). The balance between fusion and fission regulates mitochondrial morphology and function (Scott and Youle, 2010; Filadi et al., 2018). The equilibrium of fusion and fission not only determines the integrity of the mitochondrial network and maintains mitochondrial respiration but also influences several biological processes, including development, neurodegeneration, and apoptosis (Chan, 2006; Detmer and Chan, 2007). The process of mitochondrial fission is regulated by the mitochondrial fission 1 protein (FIS1) and the cytosolic dynamin 1 like protein (DNM1L), also known as DRP1 (Gottlieb and Bernstein, 2016). Mitochondrial fusion is coordinated by the outer membrane proteins mitofusin 1 (MFN1), mitofusin 2 (MFN2), and the inner membrane protein optic atrophy 1 (OPA1) (de Brito and Scorrano, 2008). Mitochondrial dysfunction and abnormal mitochondrial dynamics have been linked to a wide array of neurodegenerative diseases such as Alzheimer’s disease, Parkinson’s disease, amyotrophic lateral sclerosis, and Huntington’s disease (Su et al., 2010).

Both MFN1 and MFN2 have been reported as essential proteins for embryonic development; deletion of either *MFN1* or *MFN2* is lethal during mid-gestation in mice (Chen et al., 2003). Intriguingly, MFN2 is abundantly expressed in the brain while MFN1 expression is low, suggesting that MFN2 plays an essential role in brain development or function (Eura et al., 2003). Conditional inactivation of *MFN2* in newborn mice severely impairs cerebellar development and produces early movement defects (Chen et al., 2003). *In vitro*, MFN2 knockout leads to an aberrant fragmentation of the mitochondrial network due to a deficiency of mitochondrial fusion (Filadi et al., 2015).

Here we report disruption of mitochondrial morphology and function after ZIKV infection in human neural stem cells (NSCs) and a human glioblastoma cell line, SNB-19. We found that ZIKV infection disrupted mitochondrial dynamics, mitochondrial network structure, and function by decreasing MFN2 protein levels. Rebalancing mitochondrial dynamics by blocking mitochondrial fission in ZIKV-infected neuronal cells aided cell survival and rescued the disease phenotype, unveiling a promising therapeutic approach in controlling mitochondrial dysregulation after ZIKV infection. Together, the results strongly suggest that mitochondrial dysregulation contributes to ZIKV-mediated cell death in neuronal cells.

## Materials and Methods

### Compounds and Antibodies

The pan-caspase inhibitor emricasan was purchased from Selleckchem (S7775). The details of the antibody used in this study can be found in the section “Supplementary Material” (Supplementary Tables S1, S2).

### Cell Culture and Viruses

Glioblastoma cells, SNB-19, were maintained in RPMI-1640 medium (ATCC) supplemented with 10% fetal bovine serum (Hyclone). Osteosarcoma cells, U2OS, were maintained in McCoy’s 5a Medium (ATCC) supplemented with 10% fetal bovine serum (Hyclone). The wild-type fibroblasts (Coriell Cell Repository, GM05659) were reprogrammed and induced to pluripotent stem cells, followed by neural induction to NSCs, as described previously (Yu et al., 2014). Neural stem cells were cultured in StemPro NSC SFM (Life Technologies) containing knockout Dulbecco’s modified Eagle’s medium-F12, StemPro neural supplement, 20 ng/ml bFGF, 20 ng/ml EGF and 1X GlutaMAX on Matrigel (Corning)-coated flasks. The following viruses were used: ZIKV MR766 strain (Uganda, 1947) and ZIKV PRVABC59 strain (Puerto Rico, 2015).

### Immunocytochemistry

Cells were fixed with 4% paraformaldehyde (Sigma) for 15 min at room temperature. Samples were permeabilized with 0.25% Triton X-100 (Sigma) for 10 min and were blocked by cell staining buffer (BioLegend) for 1 h. Then, samples were incubated with primary antibody at 4°C overnight, followed by twice PBS washes and incubation with secondary antibody for 1 h at room temperature. Finally, nuclei were stained with 1 μg/ml Hoechst 33342 (Invitrogen) at room temperature for 15 min. After a final wash in PBS, 100 μl of fresh PBS was added to the cells for imaging on the IN Cell 2500 HS (GE Healthcare). Montages were generated using Fiji-ImageJ (NIH).

### Determination of Percentage of ZIKV Infection and Fluorescence Quantification

To determine the ZIKV infection rate, we took images using the IN Cell 2200 imaging system (GE Healthcare) with a 20× or 40× objective lens. Imaging detection was performed using FITC (ENV+/NS1+) and DAPI (nucleus) filter sets. Image analysis was conducted using the IN Cell Analyzer software (Version 3.7.2). With a multi-target analysis protocol, nuclei were segmented using the top-hat segmentation method with a minimum area set at 50 μm^2^ and sensitivity set to 0. ZIKV ENV staining was identified as “cells” by the analysis software and was segmented using the multi-scale top-hat algorithm. Settings for ENV detection were set to 100 μm^2^ and a sensitivity setting of 0. A filter for ENV+/NS1+ cells was set to 800 fluorescence units based on visual inspection of several wells. Infected cells were those with an average intensity greater than 800 fluorescence units. For each field in each well, the ENV+/NS1+ cells were divided by the total cell number as determined by the nuclear staining and multiplied by 100% to calculate the infection percentage per field. The final value is an average of three wells. Montages were generated using Fiji-ImageJ (NIH). To evaluate the NS1 protein expression level in the infected cell, we defined the cell contained 800–2,000 fluorescence unit of NS1 staining as a viral protein low expression cell, and the cell with NS1 intensity over 2,000 fluorescence unit as a viral protein high expression cell.

### MitoTracker Staining

On the day of the experiment, cells were live-stained with 100 μl/well of 100 nM MitoTracker^TM^ Deep Red FM (Invitrogen) in the medium at 37°C for 15 min, followed by fixation in 100 μl/well 4% paraformaldehyde (Sigma) for another 15 min and twice washed with PBS. The nuclear staining was performed by the addition of 100 μl/well of 1 μg/ml Hoechst 33342 (Invitrogen) in PBS and incubation at room temperature for 15 min. The cells were then fixed and permeabilized for subsequent immunostaining. The images were taken by the IN Cell 2200 automated fluorescence plate imaging reader (GE Healthcare) with 20× or 40× objective lens, and imaging detection was performed using DAPI (nucleus) and Cy5 (MitoTracker) filter sets. Montages were generated using Fiji-ImageJ (NIH).

### Mitochondrial Morphology Analysis

Images from the IN Cell 2200 (GE Healthcare) were loaded into FIJI (NIH) for image analysis. The Mitochondrial Network Analysis (MiNA) plugin was used in a blind analysis of randomly selected cells. Regions of interest were created manually, and the MiNA plugin quantitated the parameters described in their manuscript (Valente et al., 2017). Multiple cells per field per well were analyzed in at least three independent experiments.

### Electron Microscopy

Cells were fixed in 2% glutaraldehyde, 0.1 M cacodylate buffer, pH 7.2, for 1 h at room temperature and then stored at 4°C until transmission electron microscopy analysis was performed. The cells were washed with 0.1 M cacodylate buffer twice, post-fixed with 1% osmium tetroxide in 0.1 M cacodylate buffer for 1 h and washed again with 0.1 M cacodylate buffer twice and once with 0.1 N sodium acetate buffer before stained en bloc with 0.5% uranyl acetate in 0.1 N sodium acetate buffer for 1 h, pH 4.2. Then, cells were rewashed with 0.1 N sodium acetate buffer twice before gradual dehydration. The cells were then dehydrated in graded ethanol solutions (twice for each step of 35, 50, 70, 95%, and three times for 100%) and infiltrated overnight in pure epoxy resin (Poly/Bed 812, dodecenyl succinic anhydride (DDSA) and Nadic Methyl Anhydride (NMA) and DMP-30, Polysciences). Cells were rinsed with pure fresh resin twice, then cured in pure resin for 48 h at 55°C oven. After removing the polystyrene plates, suitable areas for thin sectioning were selected, cut out with jewelry saw, and glued onto empty resin stubs. About 70-nm thin sections were cut on an ultramicrotome (Leica EM UC6) and mounted on naked 150 mesh copper grids. The thin sections were double-stained (1:1 uranyl acetate and 70% ethanol and 1:1 lead citrate and DDH_2_O) and examined with Hitachi H-7600 transmission electron microscope, and images were taken using an AMT CCD camera (Chen et al., 2010).

### Western Blot

Cells were lysed in RIPA buffer (Enzo Life Sciences) supplemented with protease inhibitors and phosphatase inhibitor cocktail (Roche). The cell lysates were clarified by centrifugation at 20,000 × *g* for 15 min and followed by protein quantification with the BCA assay kit (Thermo Fisher Scientific). The cell lysates with similar protein concentrations were subsequently applied to Bis-Tris gels for protein separation, and the proteins were transferred from gels to polyvinylidene difluoride (PVDF) membrane by dry transfer (Thermo Fisher Scientific, iBlot 2 Gel Transfer Device). Immunoblot analysis was performed with the indicated antibodies, and the chemiluminescence signal was visualized with Luminata Forte Western HRP substrate (EMD Millipore) in the BioSpectrum system (UVP, LLC). The chemiluminescence intensities of the bands were quantified in the VisionWorks LS software (UVP, LLC).

### Establishing an Over-Expressing Mitofusin Cell Line

An improved variant of GFP, mGFP, was used tagged to the open reading frame of MFN1 (RC207184L4V, Origene), MFN2 (RC202218L4V, Origene) or control (PS100071V, Origene) in a lentiviral particle plasmid. The stable over-expression cells were generated according to the product manual. Briefly, 1 × 10^5^ SNB-19 cells were seeded in each well of a 6-well plate and incubated overnight at 37°C. Then, cells were treated with 5 μg/ml polybrene (Santa Cruz Biotechnology, Inc.) and infected with lentiviral particles at MOI = 5. After overnight incubation with the virus, cells were incubated in fresh medium for another 24 h. The stable over-expression cells were selected by 2 μg/ml puromycin medium treatment for 3 days and kept in the 0.5 μg/ml puromycin (Santa Cruz Biotechnology, Inc.) medium for three passages before the test.

### Cell Viability Assay

The ATP content assay kit (Promega or PerkinElmer Life Sciences) was applied to monitor the cell viability after ZIKV infection. Cells were seeded in white solid 96-well plates and infected with ZIKV of various MOIs as described in the result section. On the day of the experiment, 70 μl/well assay mixture (prepared according to the manufacturer’s instructions) was added to the assay plates, followed by incubation at room temperature for 10 min. The luminescence signal was determined in the luminescence mode of a Tecan multifunction plate reader.

### DePsipher Mitochondrial Potential Staining

DePsipher Mitochondrial Potential Kit (6300-100-K, R&D system) was applied to monitor the mitochondrial potential after ZIKV infection. Cells were plated in black 96-well plates with clear bottom and infected with ZIKV at MOI = 0.5, 1, or 2. On the day of the experiment, the DePsipher^TM^ solution was prepared according to the manufacturer’s instructions. Before DePsipher staining, the nuclear staining was performed by the addition of 100 μl/well of 1 μg/ml Hoechst 33342 (Invitrogen) in medium and incubation at 37°C for 15 min. After that, the medium was removed, and cells were covered with diluted DePsipher^TM^ solution, followed by incubating at 37°C for 15 min. Then, cells were washed with 100 μl of prewarmed 1× reaction buffer with Stabilizer Solution. The images were taken by the IN Cell 2200 automated fluorescence plate imaging reader (GE Healthcare) with 20× or 40× objective lens, and imaging detection was performed using DAPI (nucleus), FITC (DePsipher monomer) and TRITC (DePsipher aggregates) filter sets. Montages were generated using Fiji-ImageJ (NIH).

### Microarray Data Analysis

The GSE118305 and GSE101878 datasets were employed to predict the biological pathways in ZIKV infected cells and patients; they were downloaded from the GEO database^1^. Differentially expressed genes (DEGs) were selected by a cutoff of *p*-values <0.05 by the *T*-test. GO & KEGG pathway analysis to identify the enriched functions by DEGs using the DAVID system^2^. The *p*-value was calculated by hypergeometric distribution, and pathways with *p* < 0.05 were considered as significant. Rich factor refers to the percentage ratio of the DEGs number in the pathway and the number of all genes annotated in the pathway.

The GSE149775 dataset was download from the GEO database (see text footnote 1). For GSEA analysis of microarray data, the GSEA software was available on the GSEA-Broad Institute website. The statistical significance (nominal *p* value) of the enrichment score (ES) was estimated by running 1000 gene set permutations. The ES was normalized (NES) to account for the size of the gene set.

### Data and Statistical Analysis

All data were presented as mean ± SD with at least three independent experiments unless otherwise stated. All imaging data are presented as mean ± SD and represent data from cells in at least 10 fields from three or more independent experiments. The two-tailed unpaired Student’s test of the mean was used for single comparisons of statistical significance, and the ANOVA test with Tukey’s multiple-comparison was used for the multiple comparisons of statistical significance between experimental groups. The linear regression fit was used to analyze correlation, and R square was used to evaluate the goodness of fit.

## Results

### ZIKV Infection Impacted Mitochondria-Related Pathways

**FIGURE 1:**
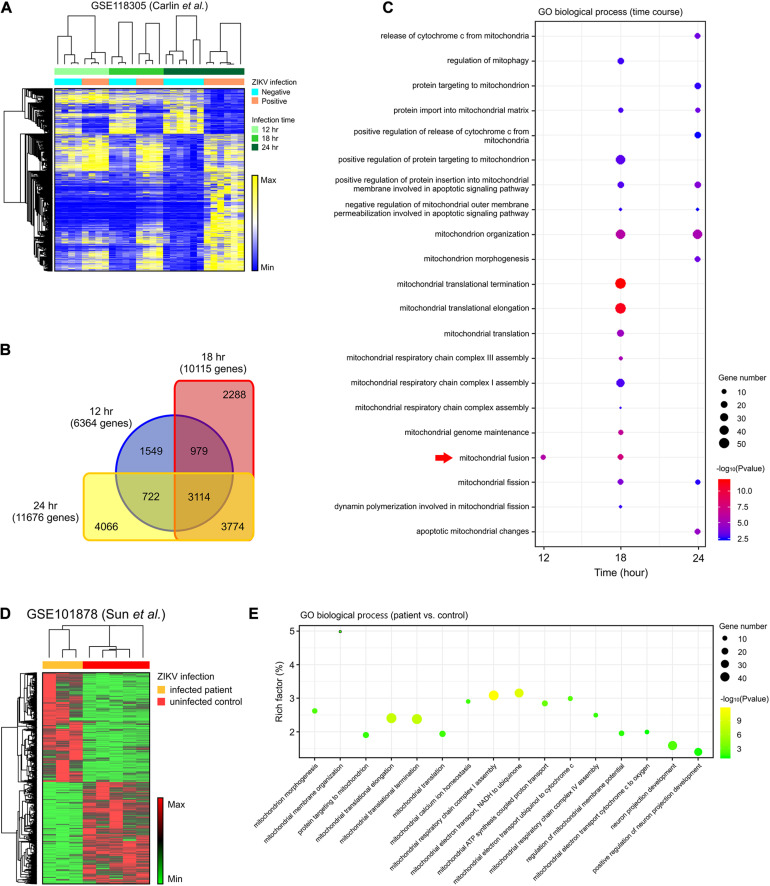
DEGs in the mitochondria-related pathways were enriched in ZIKV-infected cells. **(A)** A heatmap of all DEGs between ZIKV positive and negative cells in an RNA-seq dataset (GSE118305). **(B)** Venn diagram displaying the overlap of DEGs at each time-point in **(A)**. **(C)** Dot plot of representative mitochondria-related GO biological process enriched by DEGs at 12, 18, and 24 h post-infection in the GSE118305 data set. The *x*-axis represents the time post-ZIKV infection, and the *y*-axis represents the various pathways. Dot size represents the number of different genes, and the color indicates the *p*-value. Red arrow highlights the mitochondrial fusion-related pathway. **(D)** A heatmap of DEGs between ZIKV infected patients and uninfected control samples in an RNA-seq dataset (GSE101878). **(E)** Dot plot of representative mitochondria-related GO biological process enriched by DEGs in **(D)**. The *x*-axis represents the name of the pathway, and the *y*-axis represents the Rich factor. Dot size represents the number of different genes, and the color indicates the *p*-value.

To assess the global effects of ZIKV infection on host cells, we analyzed a publicly available genome-wide RNAseq data set (GSE118305) that contains data from ZIKV-FSS13025 infected human blood monocyte-derived macrophages (Carlin et al., 2018). We chose this dataset in order to explore a time-dependent biological reaction during ZIKV infection extensively. Compared to ZIKV-negative data, a total of 6,364; 10,115; and 11,676 DEGs were identified from ZIKV-positive cells 12, 18, and 24 h after infection, respectively (Figures 1A,B). Of these, 3,774 genes began to show differential expression at 18 h, and these changes persisted after 24 h, while 3,114 genes were differentially expressed during all time points (Figure 1B). To further investigate the cellular pathology of ZIKV infection, each set of DEGs was submitted to gene ontology (GO) pathway analysis. In addition to the viral and interferon response pathways that had been reported in the original study (Carlin et al., 2018), our analysis revealed a series of mitochondria-related biological pathways. At the earliest time point, mitochondrial fusion was significantly enriched (*p* < 0.05) (Figure 1C). At 18 h, more mitochondrial fission-fusion and function-related pathways were enriched, as well as enrichments in mitochondrial respiratory chain assembly, regulation of mitophagy, and mitochondrial-related apoptosis (Figure 1C). After 24 h of infection, there was more enrichment of mitochondrial fission rather than fusion. The analysis also revealed several mitochondria-triggered apoptotic events such as mitochondrial outer membrane permeabilization and cytochrome C release.

To further explore whether these cell-based enrichments were replicated in ZIKV-infected patients, we analyzed another RNAseq data set (GSE101878) using myeloid dendritic cells from ZIKV-infected patients (Sun X. et al., 2017). By comparing the cells of five healthy, non-infected patients (controls) with ZIKV-infected patient cells, we identified a total of 4,398 DEGs that contained 2,276 increased-expression genes and 2,122 reduced-expression genes (Figure 1D). Consistent with the *in vitro* dataset, GO pathway analysis revealed enrichment of mitochondrial morphogenesis and function pathways (Figure 1E). Additionally, the time-course data from cell-based ZIKV infection experiments in this patient-based data set also showed the enrichment of mitochondrial pathways in dendritic cells (Supplementary Figures S1B,C).

To assess whether mitochondria-related changes also occurred in ZIKV-infected neural cells, analysis was conducted on RNA-seq data set (GSE149775) of ZIKV- (MR766 and PRVABC59) infected SH-SY5Y cells (Bonenfant et al., 2020). After gene set enrichment analysis (GSEA), a significant alteration (*p* < 0.05) was observed in the GO_MITOCHONDRIAL_PROTEIN_COMPLEX annotation for both MR766- and PRVABC59-infected cells, suggesting a dysregulation and dysfunction of mitochondria in neural cells after ZIKV infection (Supplementary Figures S1H,I). Together, these data support the possibility that mitochondrial morphological and functional changes may play a critical role in ZIKV-induced pathology.

### ZIKV Infection Induced Mitochondrial Fragmentation

**FIGURE 2:**
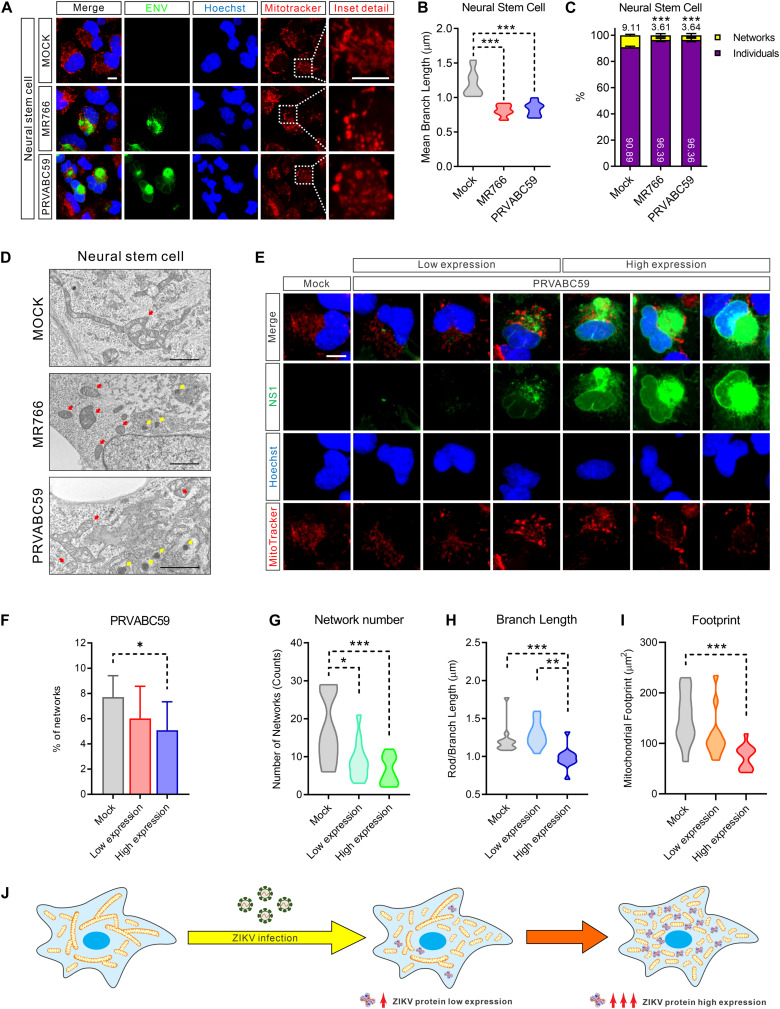
ZIKV induced mitochondria fragmentation. **(A)** Fluorescence images of NSCs 24 h after infection with MR766, PRVABC59, or mock control and stained for ZIKV ENV (green), nuclei (Hoechst, blue), and mitochondria (MitoTracker, red). Scale bar, 10 μm. **(B)** Violin plot of the mean mitochondrial branch length in **(A)** (*n* = 12). **(C)** The average percentage of the mitochondrial network and individual structures in **(A)** (*n* = 12). **(D)** Electron microscope imaging data of Mock, MR766, or PRVABC59-infected NSCs. Scale bar, 2 μm. Red arrows indicate mitochondria; yellow arrows indicate the virus particles in the rough endoplasmic reticulum. **(E)** Fluorescence images of SNB-19 infected with ZIKV PRVABC59 for 24 h and stained for ZIKV NS1 (green), nuclei (Hoechst, blue), and mitochondria (MitoTracker, red). Scale bar, 5 μm. **(F)** The average percentage of mitochondrial networks and individual structures in **(E)** (*n* = 12). **(G–I)** Violin plot representing the numbers of mitochondrial networks **(G)**, mean branch length **(H)**, and footprint **(I)** in **(E)** (*n* = 12). **(J)** A diagram shows the mitochondrial fragmentation in ZIKV-infected cells, and the fragmentation severity is positively related to the ZIKV protein expression level. All *P*-values were calculated by one-way ANOVA with Tukey’s multiple-comparison. ^∗^*p* < 0.05, ^∗∗^*p* < 0.01, and ^∗∗∗^*p* < 0.001.

To explore the interaction of ZIKV with mitochondria, we infected NSCs with either ZIKV strain MR766 (Uganda, 1947 isolate, African lineage) or PRVABC59 (Puerto Rico, 2015 clinical isolate, Asian lineage) for 24 h. MitoTracker staining of ZIKV-infected NSCs revealed abnormal mitochondrial morphology. Compared with the intact mitochondrial networks in control cells, ZIKV-infected cells showed more fragmented mitochondrial structures such as short rods and swollen puncta (Figure 2A). The mean mitochondrial branch length, the number of mitochondrial networks, and mitochondrial footprint were significantly decreased, indicating a marked reduction of mitochondrial networks and increased fragmentation (Figures 2B,C and Supplementary Figures S1D–G). We also observed the same morphological alterations in ZIKV-infected SNB-19 cells (Supplementary Figures S2A–G).

Next, we employed electron microscopy to achieve high-resolution images to assess mitochondrial network integrity. As shown in Figure 2D and Supplementary Figure S2H, more fragmented and swollen mitochondria were observed in ZIKV-infected NSCs and SNB-19 cells compared to uninfected cells. Of note, varying degrees of mitochondria structural damage and cristolysis (disappearing cristae) were found in ZIKV-infected cells. Additionally, the hive-shaped ZIKV particles accumulated in the rough endoplasmic reticulum cavity, which was juxtaposed with the fragmented mitochondria (Figure 2D, yellow arrows, and Supplementary Figure S2H). Collectively, these data indicated that ZIKV-infection disrupted healthy mitochondrial structure and caused mitochondrial fragmentation.

### Mitochondrial Fragmentation Was Related to ZIKV Protein Expression

Because ZIKV is translated as a single polypeptide that is then post-translationally processed to yield all ten viral proteins in equimolar amounts (Gerold et al., 2017), we next performed immunostaining of ZIKV NS1 protein to monitor viral protein levels in cells. While mitochondria exhibited compact filamentous network structures in uninfected NSCs, fragmented mitochondria were observed in ZIKV infected cells that related to the expression of viral protein NS1 (Figure 2E and Supplementary Figure S2I). Increased mitochondrial fragmentation is positively related to increased viral protein expression (Figure 2F and Supplementary Figure S2J).

Furthermore, fragmented mitochondria were observed surrounding the sites of viral replication in MR766 and PRVABC59-infected cells, supporting the abovementioned electron microscopy observations (Figure 2E). Concurrent with the expression of ZIKV proteins, the number of mitochondrial network structures, the mean length of the mitochondrial branches, and the footprint of total mitochondria in the cells were markedly decreased (Figures 2G–I and Supplementary Figures S2K–M). Taken together, these data indicated that the magnitude of mitochondrial fragmentation was proportional to the amount of ZIKV proteins in host cells (Figure 2J).

### Decreased MFN 2 in ZIKV-Induced Mitochondrial Fragmentation

**FIGURE 3:**
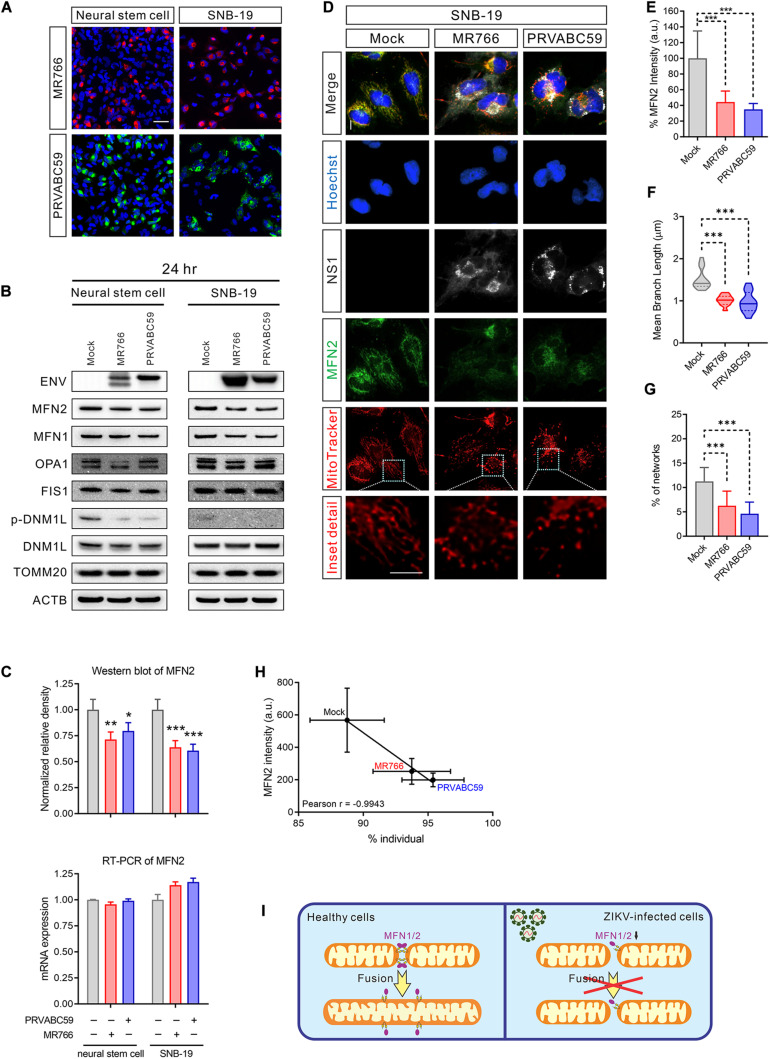
MFN2 protein level was reduced in ZIKV-infected cells. **(A)** Immunofluorescent images of ZIKV-infected NSCs and SNB-19 cells (multiplicity of infection, MOI = 2) stained for ZIKV MR766 ENV protein (red), PRVABC59 ENV protein (green), and nuclei (blue). Scale bar, 50 μm. **(B)** Western blot for MFN2, MFN1, OPA1, FIS1, DNM1L, TOMM20, and ENV from NSC and SNB-19 cell lysates 24 h after infection with Mock, MR766, or PRVABC59 (MOI = 2) Beta-actin (ACTB) is the loading control. **(C)** Quantification of MFN2 protein in the western blot in **(B)** (upper panel). Real-time PCR for MFN2 mRNA expression after 24 h of infection (lower panel). All values represent the mean ± the standard deviation (SD) (*n* ≥ 3 replicates). **(D)** Fluorescence images of SNB-19 infected with either ZIKV MR766 or PRVABC59 and stained for MFN2 (green), ZIKV NS1 (white), nuclei (blue), and mitochondria (red). Scale bar, 10 μm. **(E)** Average immunostaining intensity of MFN2 in **(D)**. **(F)** The violin plot shows the mean branch length in **(D)** (*n* = 12). **(G)** The histogram shows the percentage of the mitochondrial network in **(D)** (*n* = 12). **(H)** Pearson correlation analysis shows the relationship between the percentage of mitochondrial individual and average MFN2 intensity in **(D)**. **(I)** Graphical illustration of ZIKV-induced mitochondrial fragmentation by reduced MFN2 protein resulting in mitochondrial fusion deficient. *P*-values were calculated by two-way ANOVA with Tukey’s multiple-comparison **(C)**, one-way ANOVA with Tukey’s multiple-comparison **(E–G)**. ^∗^*p* < 0.05, ^∗∗^*p* < 0.01, and ^∗∗∗^*p* < 0.001.

Mitochondria are highly dynamic organelles; mitochondrial fusion and fission must be in equilibrium to maintain the healthy structure and function (Cao et al., 2017). To understand the cause of mitochondrial fragmentation during ZIKV infection, we hypothesized that an imbalance in mitochondrial fusion and fission proteins may lead to mitochondrial dysregulation. While only 50% of cells were infected after 24 h of virus exposure (Figure 3A and Supplementary Figure S3A), the protein, but not the mRNA, levels of MFN 2, a mitochondrial fusion-promoting protein (Rojo et al., 2002), was decreased in both ZIKV-infected NSCs and SNB-19 cells (Figures 3B,C). The reduction of MFN 1, which regulates mitochondrial outer membrane fusion (Chen et al., 2003), was only observed in SNB-19 cells but not in NSCs after ZIKV infection (Figure 3B and Supplementary Figure S3B), which might be due to the lower expression of MFN 1 in neuronal cells. As the decrease of MFN 2 in ZIKV-infected cells may be diluted in the homogenate assay due to a 50% infection rate, immunofluorescence staining was employed to detect the expression level of MFN 2 in each ZIKV-infected cell, and the results showed a weaker MFN 2 signal in ZIKV-infected cells than in uninfected cells (Figures 3D,E). Along with the decreased MFN2 protein, fragmented mitochondria were observed in ZIKV ENV-positive cells (Figures 3F–H and Supplementary Figure S3C). The results here indicated a decrease in mitochondrial fusion in ZIKV-infected cells (Figure 3I).

Protein levels of three other mitochondrial fission and fusion-related proteins, OPA1, DNM1L, and FIS1 were not significantly changed in ZIKV-infected cells (Figure 3B and Supplementary Figures S3D,E). Intriguingly, the phosphorylation of DNM1L at Ser616, a post-translational modification that activates mitochondrial fission (Gottlieb and Bernstein, 2016), was not phosphorylated in ZIKV-infected cells (Figure 3B and Supplementary Figure S3E). The dephosphorylation of DNM1L may have been due to a feedback regulation in response to the over-fragmented mitochondria to prevent even more fission. These data suggested that ZIKV infection yielded an imbalance of mitochondrial fusion and fission, leading to mitochondrial network fragmentation.

### ZIKV Infection Reduced Mitochondrial Transmembrane Potential

Typically, healthy mitochondrial morphology is reflected in the bioenergetics and function of the mitochondria (Yu T. et al., 2015). The mitochondrial transmembrane potential (delta-psi(m) or ΔΨm), generated by proton pumps (Complexes I, III, and IV), is a critical, bioenergetic parameter controlling mitochondria respiration, ATP synthesis, and the generation of reactive oxygen species (Nicholls, 2004). A drop in ΔΨm levels may induce dysfunction of mitochondria that results in a decrease in cell viability. Additionally, ΔΨm plays a key role in mitochondrial homeostasis through selective elimination of dysfunctional mitochondria. It is also a driving force for transport of ions (other than H^+^) and proteins which are necessary for healthy mitochondrial functioning (Zorova et al., 2018).

**FIGURE 4:**
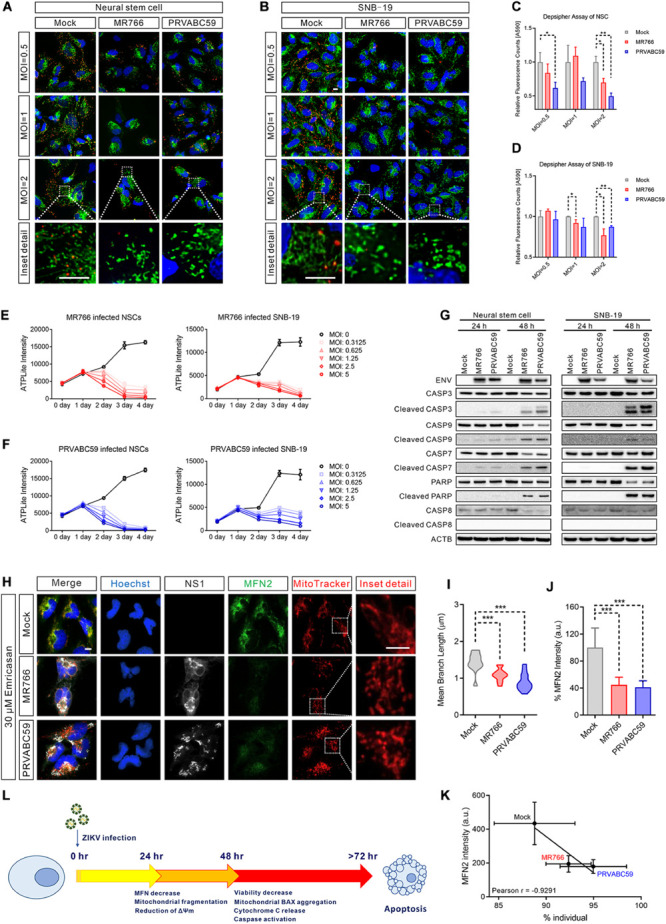
ZIKV-induced mitochondrial fragmentation proceeded apoptosis. **(A,B)** DePsipher staining images of NSCs **(A)** and SNB-19 cells **(B)** 24 h after infection with ZIKV MR766 or PRVABC59 at various MOIs (0.5, 1, and 2). Green puncta indicate the DePsipher monomers in the mitochondria. Red puncta indicate the DePsipher aggregates in the mitochondria and normal membrane potential. Scale bar, 10 μm. **(C,D)** Quantification of red puncta signal intensity in **(A)** and **(B)**, respectively. **(E)** Cell viability, as measured by intracellular ATP assay, of MR766 infected NSCs (left) and SNB-19 (right) at various MOIs (0–5). **(F)** Cell viability, as measured by intracellular ATP assay, of PRVABC59 infected NSCs (left) and SNB-19 (right) at various MOIs (0–5). **(G)** Western blot of ZIKV ENV, CASP3, cleaved CASP3, CASP9, cleaved CASP9, CASP7, cleaved CASP7, PARP, cleaved PARP, CASP8, and cleaved CASP8 of NSCs (left) or SNB-19 cells (right) at 24- or 48-h post-infection with ZIKV MR766 or PRVABC59 (MOI = 1). **(H)** Fluorescence images of SNB-19 treated with 30 μM emricasan and infected with ZIKV MR766 or PRVABC59 for 24 h. Cells were then stained for MFN2 (green), ZIKV NS1 (white), nuclei (blue) and mitochondria (red). Scale bar, 10 μm. **(I)** Violin plot of the mean branch length in **(H)** (*n* = 12). **(J)** Quantification of immunostaining intensity of MFN2 in **(H)** (*n* = 12). **(K)** Pearson correlation analysis shows the relationship between the percentage of mitochondrial individual and average MFN2 intensity in **(H)**. **(L)** A time-course schematic diagram of cells after ZIKV infection. *P*-values were calculated by two- tailed Student’s *t*-test **(C,D)** or one-way ANOVA with Tukey’s multiple-comparison test **(I,J)**. ^∗^*p* < 0.05, ^∗∗^*p* < 0.01, and ^∗∗∗^*p* < 0.001.

To determine whether the ZIKV-induced mitochondrial fragmentation disrupted mitochondrial bioenergetics, we measured the mitochondrial membrane potential using DePsipher dye, a unique cationic dye (5,5′,6,6′-tetrachloro-1,1′,3, 3′-tetraethylbenzimidazolyl-carbocyanine iodide) to indicate the loss of ΔΨm. The dyes readily enter cells and emit green fluorescence, while the dyes emit red fluorescence in healthy mitochondria as they are aggregated. When the mitochondrial membrane potential collapses, the DePsipher dye cannot accumulate within the mitochondria resulting in a reduction in red fluorescence. Thus, unhealthy mitochondria, showing primarily green fluorescence, are easily differentiated from healthy mitochondria, which show red puncta besides green fluorescence. In ZIKV-infected cells, staining with the dye DePsipher produced fewer red puncta compare with uninfected cells, indicating a reduction in mitochondrial membrane potential and impaired mitochondrial function (Figures 4A,B). In SNB-19 cells, the loss of ΔΨm was proportional to the MOI of ZIKV (started at MOI = 1) (Figure 4C). However, in NSC, infection of ZIKV PRVABC59 at MOI = 0.5 is sufficient to cause a significant decrease in membrane potential, suggesting that NSC is more sensitive to ZIKV infection than SNB-19, especially the PRVABC59 strain (Figure 4D). The cells with reduced mitochondrial membrane potential after ZIKV infection also exhibited increased mitochondrial fragmentation, fewer networks, decreased mean branch length, and a reduced mitochondrial footprint (Supplementary Figures S4A–C). The results revealed impaired mitochondrial function after ZIKV infection combined with mitochondrial fragmentation.

### ZIKV-Induced Mitochondrial Fragmentation Occurred Prior to Apoptosis

Although mitochondrial fragmentation has been shown to precede cell death, it remains unclear whether it is sufficient to activate the cell death pathway (James and Martinou, 2008). To examine whether mitochondrial fragmentation contributes to ZIKV-induced cell death, we determined the temporal relationship of mitochondrial fragmentation and cell death after ZIKV infection in NSCs and SNB-19 cells using a cell viability assay measuring ATP content in cells. Compared with uninfected controls, intracellular ATP levels began to decrease at 48 h post-infection in both the MR766 and PRVABC59 ZIKV strains (Figures 4E,F). As several studies have shown that ZIKV infection results in apoptosis of infected cells (Ghouzzi et al., 2016; Xu et al., 2016; Zhang et al., 2016; Devhare et al., 2017; Zhu et al., 2017), we also measured the caspase activity in the ZIKV-infected cells. Western blots showed significant activation of caspase-3, -9, -7, and poly (ADP-ribose) polymerase (PARP) (which indicated intrinsic apoptotic pathway activation), but not caspase-8 (which is involved in the extrinsic apoptotic signaling pathways), after 48 h of infection (Figure 4G). We also observed the release of cytochrome C from mitochondria and the aggregation of the apoptosis regulator BCL2-associated X (BAX) on the mitochondrial outer membrane 48 h post-infection (Supplementary Figures S4D,E), indicating that the intrinsic apoptotic pathway played a critical role in ZIKV-induced cell death.

Subsequently, we used the pan-caspase inhibitor emricasan to block cell death caused by ZIKV infection (Hoglen et al., 2004), which validated results from our previous study (Xu et al., 2016). However, mitochondrial fragmentation was still observed in the emricasan treated cells after ZIKV infection (Figures 4H,I and Supplementary Figure S4F). This result suggested that ZIKV-induced mitochondrial fragmentation was prior to or independent of caspase activation and apoptotic cell death in ZIKV-infected cells. Regardless of the presence of emricasan, MFN 2 was significantly decreased in ZIKV-infected cells, and the reduced MFN 2 level correlated with an increased mitochondrial fragmentation (Figures 4J,K and Supplementary Figure S4G). These data suggested that mitochondrial fragmentation could precede or be independent of apoptosis (Figure 4L).

### Blocking Mitochondrial Fragmentation Increased Cell Survival After ZIKV Infection

**FIGURE 5:**
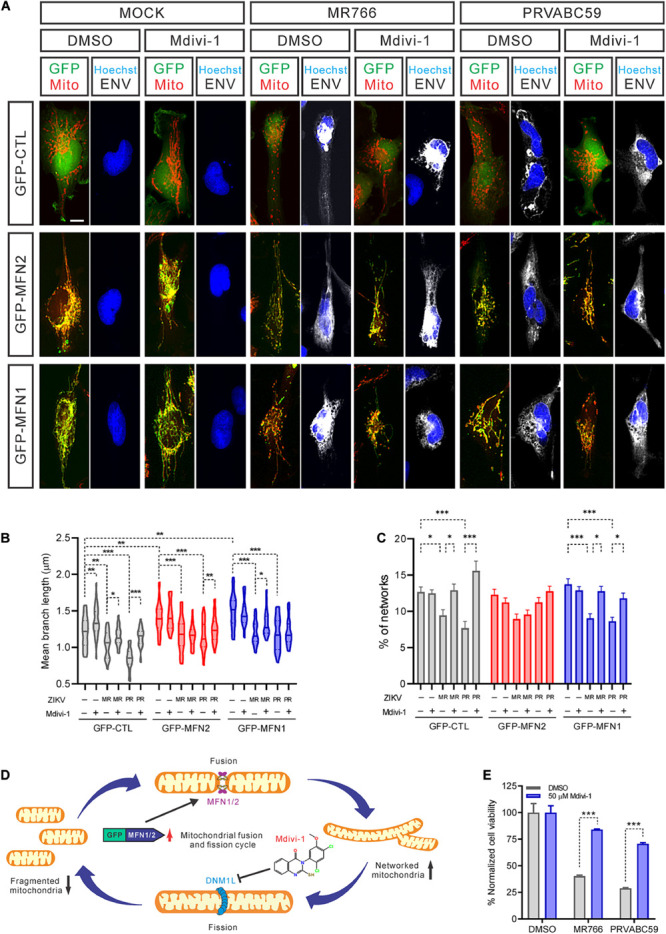
Inhibiting mitochondrial fragmentation increased cell survival after ZIKV infection. **(A)** Fluorescence images of SNB-19 over-expressing GFP-MFN1 or GFP-MNF2 (green) treated with 50 μM Mdivi-1 and infected with ZIKV for 24 h. Cells were then stained for ZIKV ENV (white), nuclei (blue), and mitochondria (red). Scale bar, 10 μm. **(B)** The violin plot shows the mean branch length in **(A)** (*n* = 18). **(C)** The histogram shows the percentage of mitochondrial networks in **(A)** (*n* = 18). **(D)** A diagram of mitochondrial fusion and fission cycle and our strategy to rebalance mitochondrial dynamics, increase mitochondrial fusion by over-expression MFNs and/or decrease mitochondrial fission by Mdivi-1 treatment, which blocks DNM1L activity. **(E)** SNB-19 cell viability after 48 h infection with ZIKV at MOI = 5 in the presence of 50 μM Mdivi-1 or DMSO. The values represent mean ± SD (*n* ≥ 3 replicates). All *P*-values were calculated by two-way ANOVA with Tukey’s multiple-comparison. ^∗^*p* < 0.05, ^∗∗^*p* < 0.01, and ^∗∗∗^*p* < 0.001.

Mitochondrial fission and fusion are balanced to maintain healthy mitochondrial morphology and function in cells. To further explore if blocking mitochondrial fragmentation would help increase cell survival after ZIKV infection, we over-expressed both MFN1 and MFN2 to restore normal fusion and treated cells with Mdivi-1 to inhibit DNM1L activity, reducing mitochondrial fission. Both over-expression of MFNs and treatment with Mdivi-1 produced a hyperconnected mitochondrial network in uninfected cells (Figure 5A). However, a combination of over-expressed MFNs and Mdivi-1 treatment did not further change the mitochondrial structure (Figure 5A). In uninfected control cells, Mdivi-1 treatment significantly increased the mean branch length of mitochondria, but not the numbers of individual mitochondria (Figures 5B,C). After ZIKV infection, Mdivi-1 treatment attenuated mitochondrial fragmentation in control SNB-19 cells. We also observed that in MFN1 or MFN2 over-expressing cells, Mdivi-1 moderately increased mitochondrial branch length and the number of mitochondria in the networks. Although ZIKV infection still shortened mitochondrial branch length in cells overexpressing MFN1 or MFN2, the range of branch lengths in these cells was similar to uninfected controls (Figure 5B). The mitochondrial networks were only spared in MFN2 and not MFN1 overexpressing cells after ZIKV infection (Figure 5C). These data suggested that either inhibition of mitochondrial fission (by inhibiting DNM1L) or induction of mitochondrial fusion (by over-expression of MFN2) could counteract the mitochondrial fragmentation caused by ZIKV infection (Figure 5D).

In addition, we found that Mdivi-1 treatment alone prevented a decrease in cell viability after 48 h of infection without affecting the rate of infection (Figure 5E and Supplementary Figure S5A). Together with our previous data, this result suggested that mitochondrial fragmentation precedes ZIKV-induced cell death. However, we did not find that over-expression of MFN2 or MFN1 protein rescued cell death caused by ZIKV infection (Supplementary Figure S5B). This phenomenon could be due to increased mitochondrial fusion that does not effectively reduce the cytochrome C release from ZIKV-promoted mitochondrial fission, leading to the apoptosis (Suen et al., 2008). Collectively, these data indicated that maintenance of normal mitochondrial dynamics by inhibiting mitochondrial fission corrected ZIKV-induced mitochondrial changes and increased cell survival after ZIKV infection. This finding hints at a potential therapeutic intervention – inhibition of mitochondrial fission in neural cells – as a treatment for ZIKV infection.

## Discussion

The association between ZIKV infection and microcephaly has motivated research towards the development of neurotherapeutic interventions (Xu et al., 2016; Yang et al., 2018). ZIKV preferably infected iPSC-derived human neural progenitor cells with high efficiency resulting in cell death compared to differentiated primary neurons (Tang et al., 2016). ZIKV also impaired the growth of iPSC-derived human neurospheres and brain organoids (Garcez et al., 2016; Qian et al., 2016). In mouse models, ZIKV infection caused neurodevelopmental abnormalities (Lazear et al., 2016; Miner et al., 2016; Rossi et al., 2016; Schuler-Faccini et al., 2016; Yang et al., 2018). Of note, several development-related pathways were enriched in our analysis of ZIKV-infected cells (*p* < 0.05), including stem cell population maintenance, regulation of cell differentiation, forebrain morphogenesis, and neuron differentiation (Supplementary Figure S1A).

Abnormal morphology and dysfunction in mitochondria have been linked to many neurological diseases (Leshinsky-Silver et al., 2002; Mattson et al., 2008; Condie et al., 2010; Falk, 2010). MFN proteins have been shown to be essential for maintaining healthy neuronal function and are dysregulated in several neurological diseases like Parkinson’s disease (Brooks et al., 1999; Betarbet et al., 2006; Tanaka et al., 2010; Lee et al., 2012), Alzheimer’s disease (Wang et al., 2009; Manczak et al., 2011; Chen et al., 2016), Huntington’s disease (Shirendeb et al., 2011), and Charcot-Marie-Tooth disease type 2A (Verhoeven et al., 2006; Calvo et al., 2009; Song et al., 2013). Abnormalities of other critical mitochondrial dynamics parameters were also reported in some neurological disorders. The abnormal activation of DNM1L has been reported in Alzheimer’s disease (Cho et al., 2009) and Huntington’s disease (Shirendeb et al., 2011; Song et al., 2011), both of which promoted mitochondrial fission and damaged neurons. Suppressing mitochondrial fission by inhibiting DNM1L activity or overexpressing MFN2 restored healthy mitochondrial dynamics, preserved ATP, and prevented cell death (Wang et al., 2009). Notably, abnormal mitochondrial morphology and dysfunction such as giant individual mitochondria were also reported in newborns with microcephaly (Suzuki and Rapin, 1969; Waterham et al., 2007).

Mitochondria are dynamic organelles, continuously undergoing cycles of fusion, and fission to maintain a wide range of biological functions (Twig et al., 2008). Several studies have reported that mitochondrial dynamics can be altered during infections with pathogens such as viruses, bacteria, and parasites, leading to changes in mitochondrial morphology (Kim et al., 2013; Stavru et al., 2013; Gorbunov et al., 2015; Ding et al., 2017; Escoll et al., 2017). In previous reports, MFN2-knockout cells displayed mitochondrial spheres or ovals of widely different sizes, including some with a diameter several-fold larger than wild type mitochondrial tubules (Filadi et al., 2018), similar to the mitochondrial phenotype of infants with microcephaly (Suzuki and Rapin, 1969). Mitochondria trafficking in neurons is mediated by MFN2, and disruption of this function induces axon degeneration (Baloh et al., 2007; Misko et al., 2012). In this study, the lower levels of MFN2 after ZIKV infection implies a role for mitochondrial dysregulation in ZIKV-mediated microcephaly. Although over-expression of MFN2 in ZIKV-infected cells helped to prevent the pathological changes in mitochondrial morphology, it failed to rescue cells from the ZIKV-induced cell death. Interestingly, a mitochondrial fission inhibitor, Mdivi-1, effectively reversed ZIKV-induced mitochondrial morphological and functional changes, rescuing cells from apoptotic cell death. We hypothesize that the over-expression of MFN2 failed to rescue ZIKV infected cells from cell death because the release of cytochrome C was not blocked by MFN2 over-expression. In contrast, inhibition of mitochondrial fission by Mdivi-1 not only prevented mitochondrial fragmentation by rebalancing mitochondrial dynamics but also prevented the release of cytochrome C, which usually occurs during mitochondrial fission (Suen et al., 2008). Thus, rebalancing mitochondrial fission and fusion may be one strategy to mitigate the cellular pathology caused by ZIKV infection.

In agreement with a previous report (Tang et al., 2016), the mRNA expression levels of MFN1 and MFN2 were not changed in ZIKV-infected iPS cell-derived cortical neural progenitor cells compared with the control cells. Our data indicated that the reduction of MFN2 protein occurred independently without changes in the mRNA levels. However, a reduction of MFN2 mRNA was observed in the myeloid dendritic cells from ZIKV-infected patients, as well as the dendritic cells 24 h after infection *in vitro* (Carlin et al., 2018), implying differential regulation of MFN2 expression in different types of host cells during ZIKV infection (Supplementary Figures S3F,G). The MFN1 and MFN2 (MFNs) mRNA levels remain unchanged in neuronal cells. Therefore, the protein-level decreases of MFNs are most likely due to an increase in MFN protein degradation or a decrease in MFN protein synthesis rather than transcriptional repression; additionally, reasons for the changes in MFN levels may vary depending on cell type.

Other *Flaviviridae* viruses have been reported to modulate mitochondrial proteins and function such as hepatitis C virus, West Nile virus, and dengue virus (DENV) (Li et al., 2005; Piccoli et al., 2006; Ramanathan et al., 2006; Aguirre et al., 2017; Sun B. et al., 2017). In our study, we observed significant fragmentation of mitochondrial networks in both MR766- and PRVABC59-infected NSCs and glioblastoma SNB-19 cells. The degree of mitochondrial fragmentation correlated positively with ZIKV protein expression levels. Consistent with the report by Garcez et al. (2016), some ZIKV-infected cells exhibited mitochondrial swelling. Interestingly, round and giant neuronal mitochondria were observed in microcephalic infants (Suzuki and Rapin, 1969). Chatel-Chaix et al. (2016) observed mitochondrial elongation in Huh7 cells after ZIKV infection, possibly due to its heterogeneous effects on different cell types. The variation of changes in mitochondrial morphology observed in ZIKV infection was also present in DENV research; Yu C.Y. et al. (2015) demonstrated DENV cleaved MFN1 and MFN2 via its NS2B-NS3 protease to impair mitochondrial fusion. Others reported DENV-induced mitochondrial elongation through inhibition of DNM1L-triggered mitochondrial fission via NS4B (Chatel-Chaix et al., 2016) or via a combination of NS4B and NS3 (Barbier et al., 2017). Several ZIKV proteins (NS4A, NS4B, and NS5) have been reported to affect interferon-related signaling pathways by targeting the mitochondrial antiviral signaling protein (MAVS) to evade the host antiviral response (Grant et al., 2016; Wu et al., 2017; Ma et al., 2018). These reports have implicated the disruption of mitochondria function by ZIKV infection.

ZIKV-induced apoptosis (Ghouzzi et al., 2016; Souza et al., 2016; Tang et al., 2016; Zhang et al., 2016; Oh et al., 2017) was reported to be mediated by TP53 (Ghouzzi et al., 2016). In our study, ZIKV-induced cell death was observed only after 48 h of ZIKV infection, while mitochondrial fragmentation was present at 24 h post-infection. Concurrently, we also observed the loss of mitochondrial transmembrane potential, indicating disrupted mitochondrial function after ZIKV infection. It has been reported that over-expression of ZIKV NS4A in a fission yeast led to cell death, which occurred in part by the induction of intracellular oxidative stress (Li et al., 2017). In this work, we demonstrated mitochondrial fragmentation as an early event that preceded cell death in NSCs infected by ZIKV. However, the changes in mitochondria after ZIKV infection may vary in different cell types and may have different effects on cell death events.

In summary, our work uncovered mitochondrial fragmentation caused by ZIKV infection preceding cell death. The MFN2 protein level in mitochondria was reduced in the ZIKV infected cells, and over-expression of MFN2 in these cells rescued the changes in morphology and function of the mitochondria. Mdivi-1, the mitochondrial fission inhibitor targeting DNM1L, also blocked mitochondrial fragmentation and cell death caused by ZIKV infection. The reduction of the MFN2 level caused a deficiency in mitochondrial fusion, resulting in the fragmentation of the mitochondria. Therefore, rebalancing mitochondrial dynamics may serve as a therapeutic strategy for drug development to treat neuronal complications of ZIKV infection. Future work in whole animal models will be needed to validate the restoration of mitochondrial dynamics as a strategy to reduce neuronal damage after ZIKV infection.

## Data Availability Statement

Publicly available datasets were analyzed in this study. This data can be found here: https://www.ncbi.nlm.nih.gov/geo/query/acc.cgi?acc=GSE118305 and https://www.ncbi.nlm.nih.gov/geo/query/acc.cgi?acc=GSE149775.

## Author Contributions

SY, NS, GM, HS, HT, and WZ conceived the research and designed the study. SY, KG, EL, MX, Y-SC, FS, and NV performed the experiments. SY and KG analyzed the data. SY, KG, EL, Y-SC, NS, HT, and WZ wrote, reviewed, and edited the manuscript. All authors contributed to the article and approved the submitted version.

## Conflict of Interest

The authors declare that the research was conducted in the absence of any commercial or financial relationships that could be construed as a potential conflict of interest.

## Footnotes

1 www.ncbi.nlm.nih.gov/geo/

2 https://david.ncifcrf.gov/
