# Supplementary material for: Zika Virus-Induced Neuronal Apoptosis via Increased Mitochondrial Fragmentation
Source: Front Microbiol. 2020 Dec 23;11:598203. doi: 10.3389/fmicb.2020.598203 (PMC7785723; doi:10.3389/fmicb.2020.598203)
Supplement: Supplementary file 1 [file Data_Sheet_1.docx]

**Supplemental Table S1. Primary Antibodies, catalog numbers, and sources.**

| **Primary antibodies** | **Catalog number** | **Company** |
| --- | --- | --- |
| Anti-zika envelope mAb^1^ | BF-1176-56 | BioFront Technologies |
| Anti-zika ns1 mAb | BF-1225-06 | BioFront Technologies |
| Mitofusin-2 (D1E9) rabbit mAb | 11925 | Cell Signaling Technology |
| Mitofusin-1 (D6E2S) rabbit mAb | 14739 | Cell Signaling Technology |
| Tom20 (D8T4N) rabbit mAb | 42406 | Cell Signaling Technology |
| Β-Actin (8H10D10) Mouse mAb | 3700 | Cell Signaling Technology |
| OPA1 (D7C1A) rabbit mAb | 67589 | Cell Signaling Technology |
| Phospho-DRP1 Ser616 (D9A1) rabbit mAb | 4494 | Cell Signaling Technology |
| DRP1 (4E11B11) Mouse mAb | 14647 | Cell Signaling Technology |
| Caspase-3 (8G10) rabbit mAb | 9665 | Cell Signaling Technology |
| Cleaved caspase-3 (asp175) antibody | 9661 | Cell Signaling Technology |
| Caspase-9 (C9) Mouse mAb | 9508 | Cell Signaling Technology |
| Cleaved Caspase-9 Asp330 (D2D4) rabbit mAb | 7237 | Cell Signaling Technology |
| Caspase-8 (D35G2) rabbit mAb | 4790 | Cell Signaling Technology |
| Cleaved Caspase-8 Asp384 (11G10) Mouse mAb | 9748 | Cell Signaling Technology |
| Caspase-7 antibody | 9492 | Cell Signaling Technology |
| Cleaved caspase-7 asp198 antibody | 9491 | Cell Signaling Technology |
| PARP (46D11) rabbit mAb | 9532 | Cell Signaling Technology |
| Cleaved PARP Asp214 (D64E10) XP® rabbit mAb | 5625 | Cell Signaling Technology |
| Fis1 antibody (b-5) | sc-376447 | Santa Cruz Biotechnology |

^1^mAb = monoclonal antibody.

**Supplemental Table S2. Secondary Antibodies, catalog numbers, and sources**

| **Secondary antibodies** | **Catalog number** | **Company** |
| --- | --- | --- |
| Anti-mouse IgG H+L, F ab' 2 Fragment Alexa Fluor® 488 Conjugate | 4408 | Cell Signaling Technology |
| Anti-rabbit IgG H+L, F ab' 2 Fragment Alexa Fluor® 594 Conjugate | 8889 | Cell Signaling Technology |
| Anti-mouse IgG, HRP-linked Antibody | 7076 | Cell Signaling Technology |
| Anti-rabbit IgG, HRP-linked Antibody | 7074 | Cell Signaling Technology |
