# Supplementary material for: Zika Virus-Induced Neuronal Apoptosis via Increased Mitochondrial Fragmentation
Source: Front Microbiol. 2020 Dec 23;11:598203. doi: 10.3389/fmicb.2020.598203 (PMC7785723; doi:10.3389/fmicb.2020.598203)
Supplement: Supplementary file 2 [file Data_Sheet_2.docx]

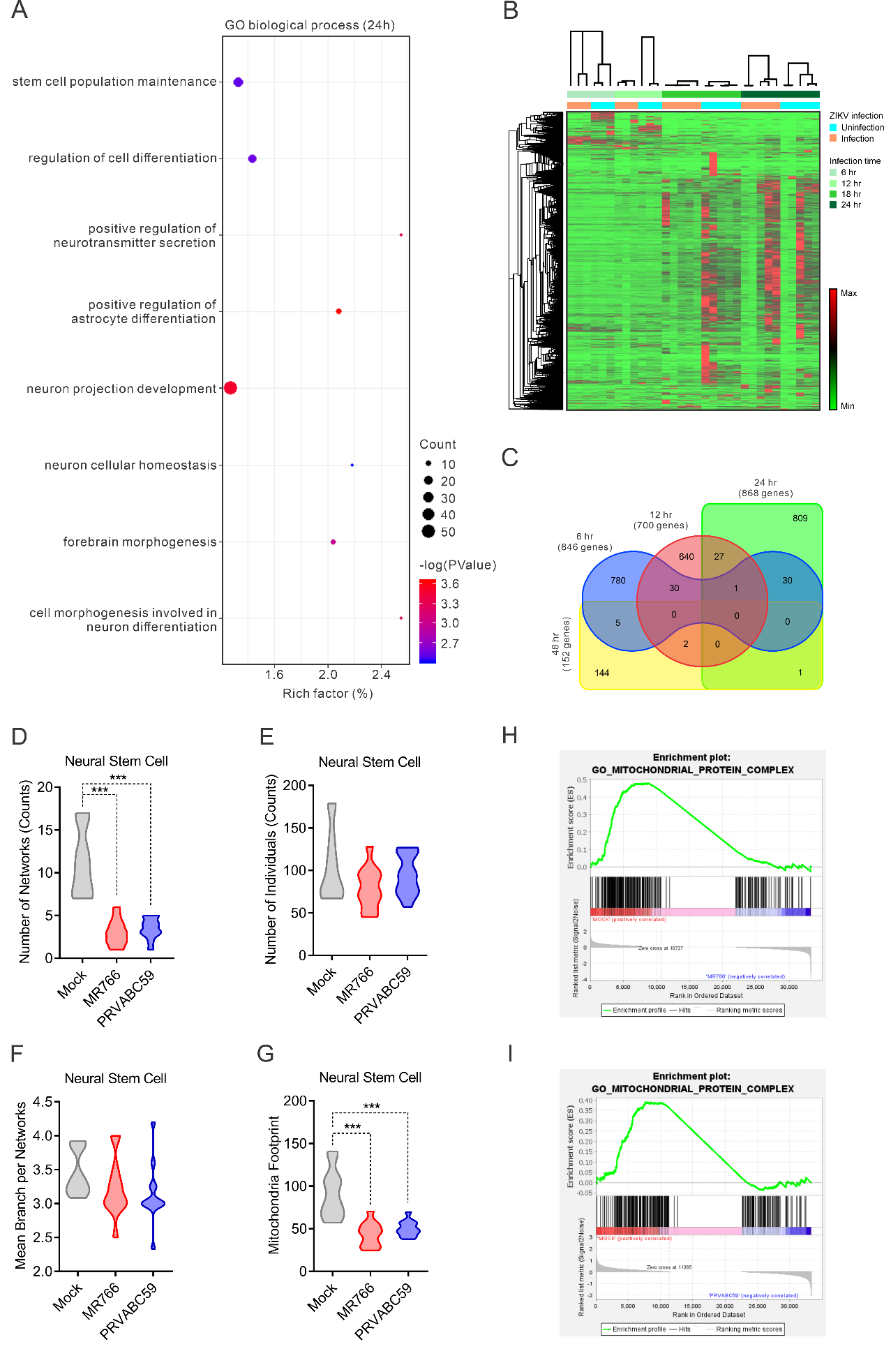


**Supplementary Figure S1. ZIKV infection impacts mitochondrial dynamics**

(**A**) Dot plot of representative neuronal development-related GO biological process enriched by DEGs at 24 hours post-infection in GSE118305. The y-axis represents the name of the pathway, and the x-axis represents the Rich factor. Dot size represents the number of different genes and the color indicates the p-value. (**B**) A heatmap of all DEGs for ZIKV infected and uninfected dendritic cells with time-course infection in an RNA-seq dataset (GSE101878). (**C**) Venn diagram displaying the overlap of DEGs at each time-point in **A**. (**D-G**) Violin plot of the number of mitochondrial networks (**D**), number of mitochondrial individuals (**E**), mean branch per networks (**F**) and mitochondrial footprint (**G**) in **Figure 2A** (n=12). (**H**) GSEA plot showing an enrichment of gene signatures associated with MITOCHONDRIAL PROTEIN COMPLEX between MOCK and MR766-infected SH-SY5Y cells. (**I**) GSEA plot showing an enrichment of gene signatures associated with MITOCHONDRIAL PROTEIN COMPLEX between MOCK and PRVABC59-infected SH-SY5Y cells. All significance was analyzed by one-way ANOVA with Tukey’s multiple-comparison. *, p<0.05, **, p<0.01, ***, p < 0.001.


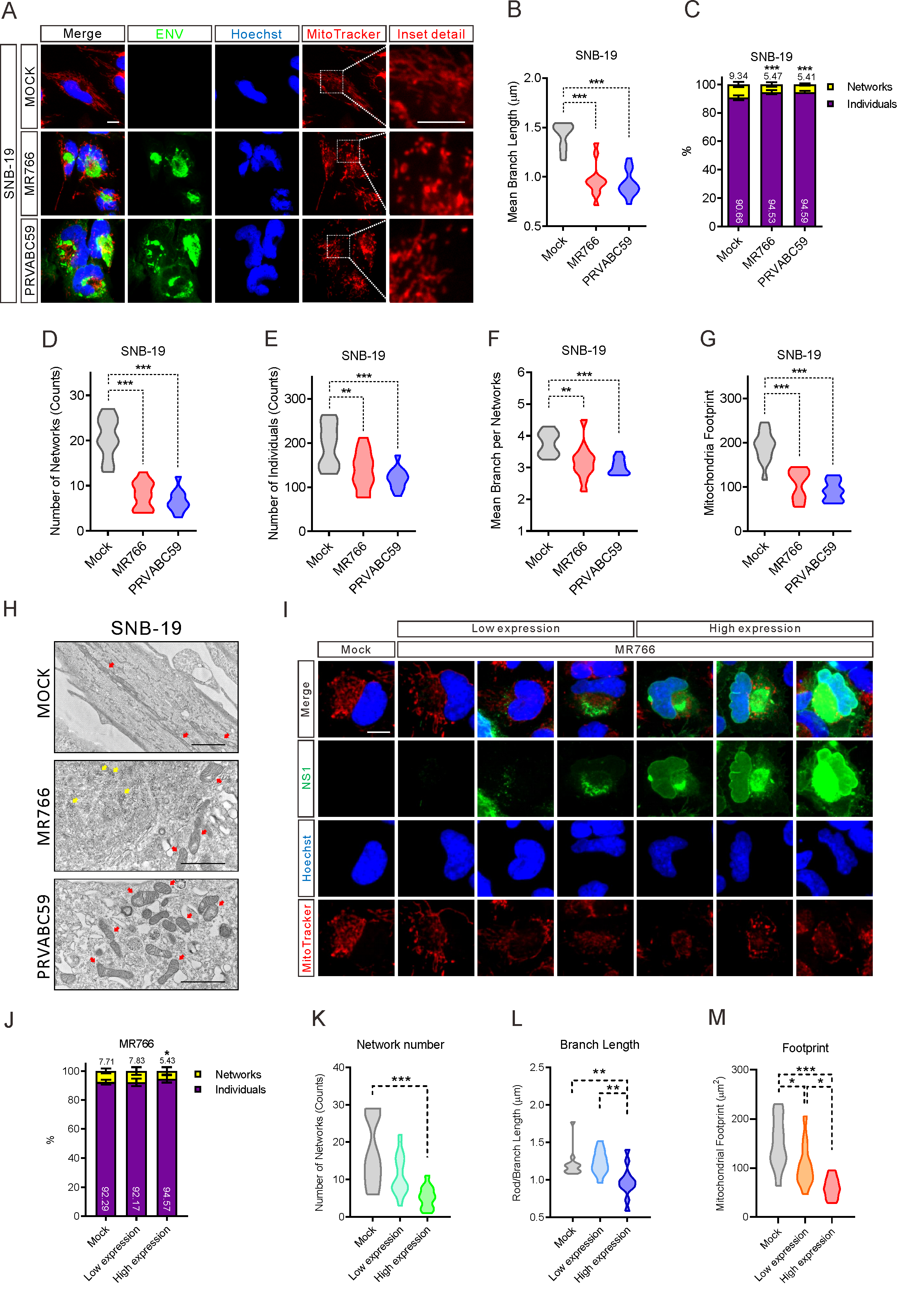


**Supplementary Figure S2. ZIKV induces mitochondria fragmentation**

(**A**) Fluorescence images of SNB-19 cells 24 hours after infection with Mock (control), MR766 or PRVABC59 and stained for ZIKV ENV (green), nuclei (blue) and mitochondria (red). Scale bar, 10 µm. (**B**) Violin plot of the mean mitochondrial branch length in **A** (n=12). (**C**) Average percentage of mitochondrial network and individual structures in **A** (n=12). (**D-G**) Violin plot of the number of mitochondrial networks (**D**), number of mitochondrial individuals (**E**), mean branch per networks (**F**) and mitochondrial footprint (**G**) in **A** (n=12). (**H**) Electron microscope imaging data of Mock, MR766 or PRVABC59-infected SNB-19 cells. Scale bar, 2 µm. Red arrows indicate mitochondria; yellow arrows indicate the viral particles in the rough endoplasmic reticulum. (**I**) Fluorescence images of SNB-19 infected with ZIKV MR766 for 24 hours and stained for ZIKV NS1 (green), nuclei (blue) and mitochondria (red). Scale bar, 5 µm. (**J**) Average percentage of mitochondrial networks and individual structures in **I** (n=12). (**K-M**) Violin plot representing the numbers of mitochondrial networks (**K**), mean branch length (**L**), and footprint (**M**) in **I**, (n=12). All significance was analyzed by one-way ANOVA with Tukey’s multiple-comparison. *, *p*<0.05, **, *p*<0.01, ***, *p* < 0.001.


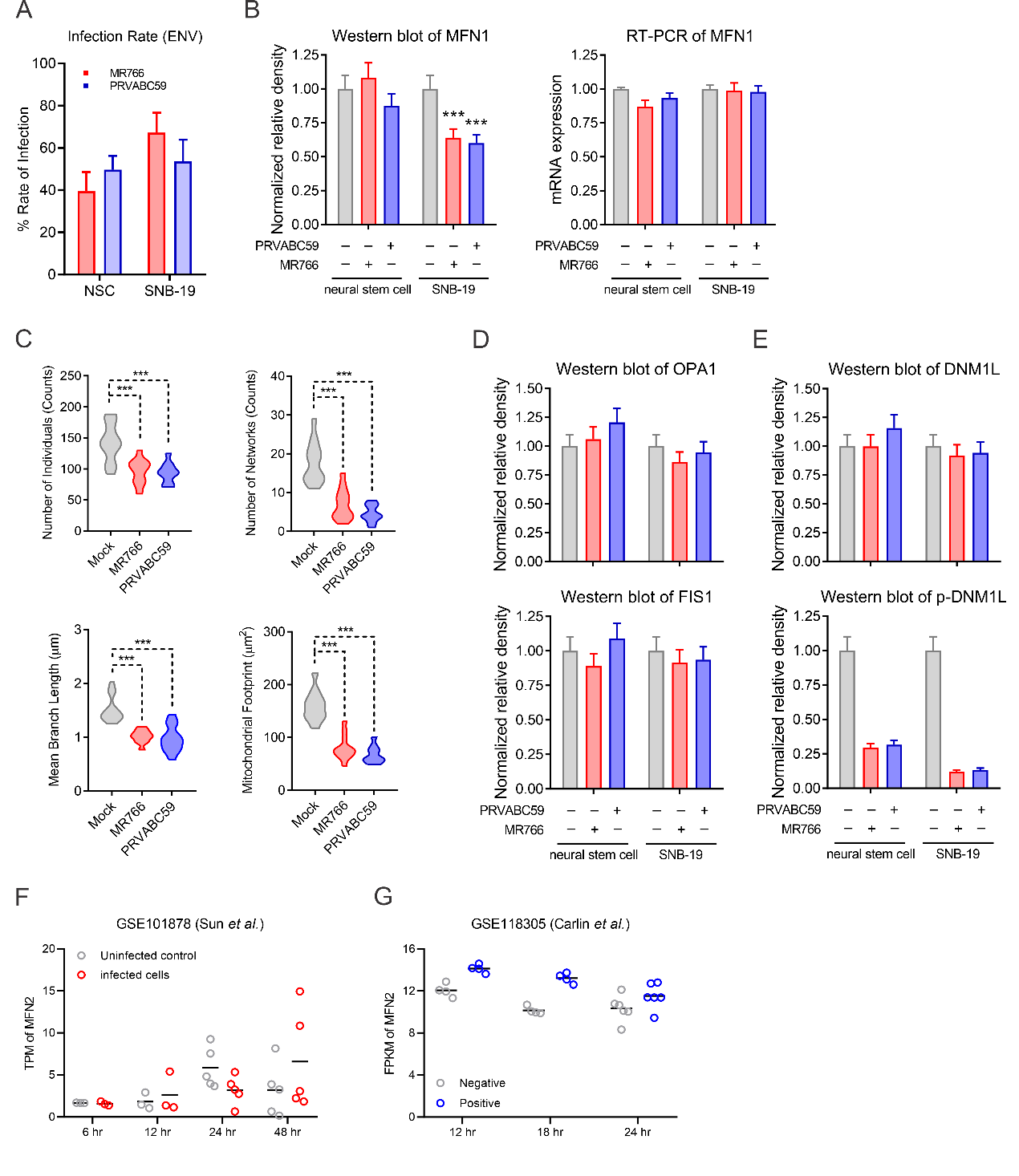


**Supplementary Figure S3. MFN2 is involved in ZIKV-induced mitochondrial fragmentation.**

(**A**) The infection rate of NSCs and SNB-19 cells in **Figure 3A**. (**B**) Quantification of MFN1 protein using western blot in **Figure 3B** (left panel). Real-time PCR for MFN1 mRNA expression after 24 hour infection (right panel). (**C**) Violin plot representing the numbers of mitochondrial individuals (left upper panel), number of networks (right upper panel), mean branch length (left lower panel), and footprint (right lower panel) in **Figure 3D**, (n=12). (**D**) Quantification of OPA1 protein (upper panel) and FIS1 (lower panel) in the western blot in **Figure 3B**. (**E**) Quantification of DNM1L protein (upper panel) and phosphorylated DNM1L ser616 (lower panel) in the western blot in **Figure 3B**. (**F**) Time-course data of MFN2 TPM between ZIKV-infected cells and control in RNA-seq data GSE101878. (**G**) Time-course data of MFN2 FPKM between ZIKV-positive and negative cells in RNA-seq data GSE118305.All values represent mean ± SD (n≥3 replicates). All Significance was analyzed by one-way ANOVA with Tukey’s multiple-comparison. *, *p*<0.05, **, *p*<0.01, ***, *p* < 0.001.


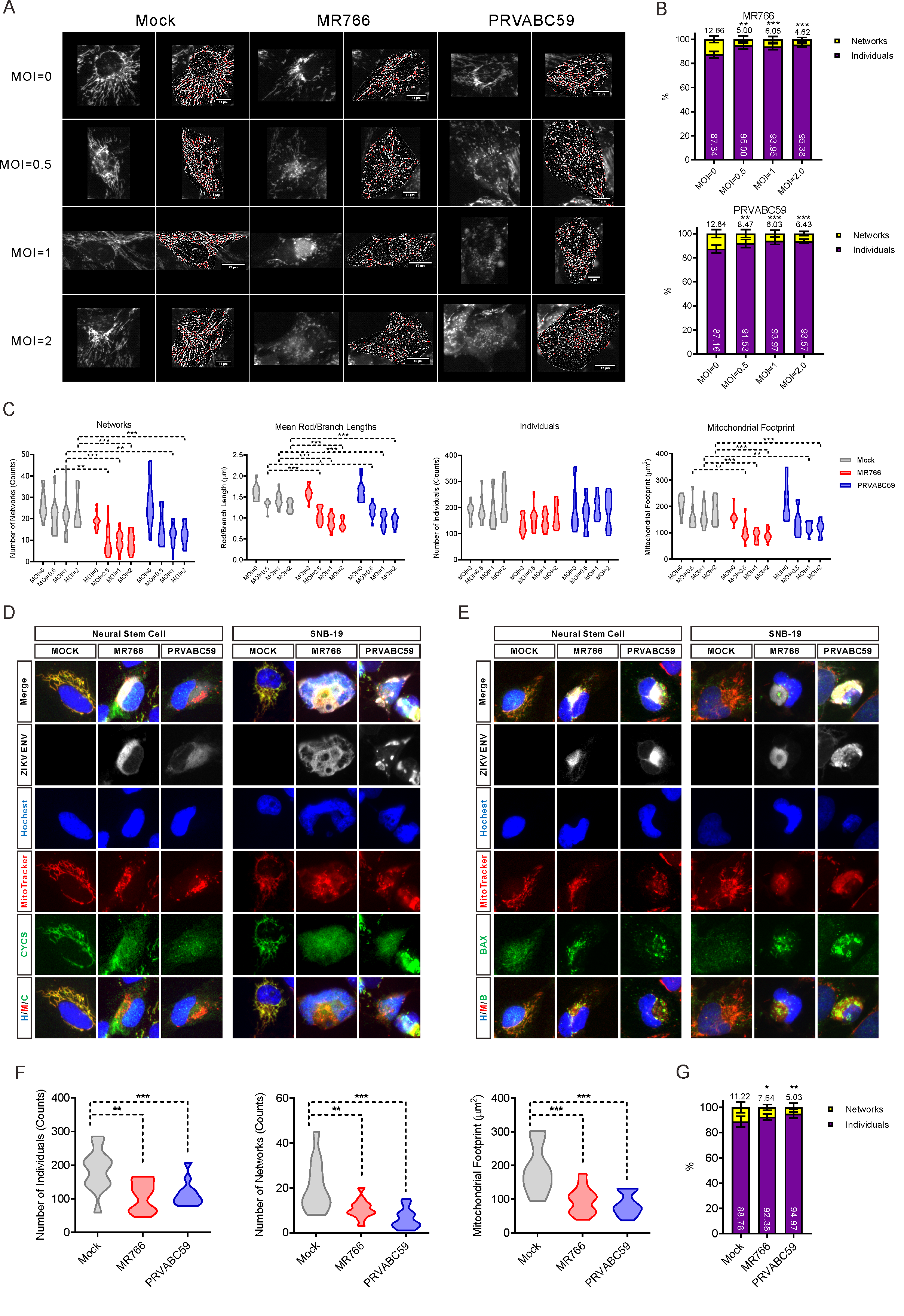


**Supplementary Figure S4. ZIKV-induced mitochondrial fragmentation precedes apoptosis.**

(**A**) Mitochondrial structure analysis of DePsipher staining images of NSCs 24 hours after infection with ZIKV MR766 or PRVABC59 at various MOIs (0, 0.5, 1, 2) by ImageJ plug-in, MiNA. (**B**) Percentage of mitochondrial network and individual structure after infection of MR766 (upper panel) and PRVABC59 (lower panel) in **A** (n=12). (**C**) Violin plot representing the numbers of mitochondrial networks (the first panel), mean branch length (the second panel), number of individuals (the third panel) and footprint (the last panel) in **A**, (n=12). (**D**) Fluorescence images of NSC (left panel) and SNB-19 (right panel) infected with ZIKV MR766 or PRVABC59 for 48 hours. Cells were then stained for CYCS (green), ZIKV ENV (white), nuclei (blue) and mitochondria (red). (**E**) Fluorescence images of NSC (left panel) and SNB-19 (right panel) infected with ZIKV MR766 or PRVABC59 for 48 hours. Cells were then stained for BAX (green), ZIKV ENV (white), nuclei (blue) and mitochondria (red). (**F**) Violin plot representing the numbers of mitochondrial individuals (left panel), number of networks (middle panel) and footprint (right panel) in **Figure 4H**, (n=12). (**G**) Percentage of mitochondrial network and individual structure in **Figure 4H**, (n=12). The significance was analyzed by one-way ANOVA with Tukey’s multiple-comparison (**B**, **F**, **G**) or two-way ANOVA with Tukey’s multiple-comparison (**C**). *, *p*<0.05, **, *p*<0.01, ***, *p* < 0.001.


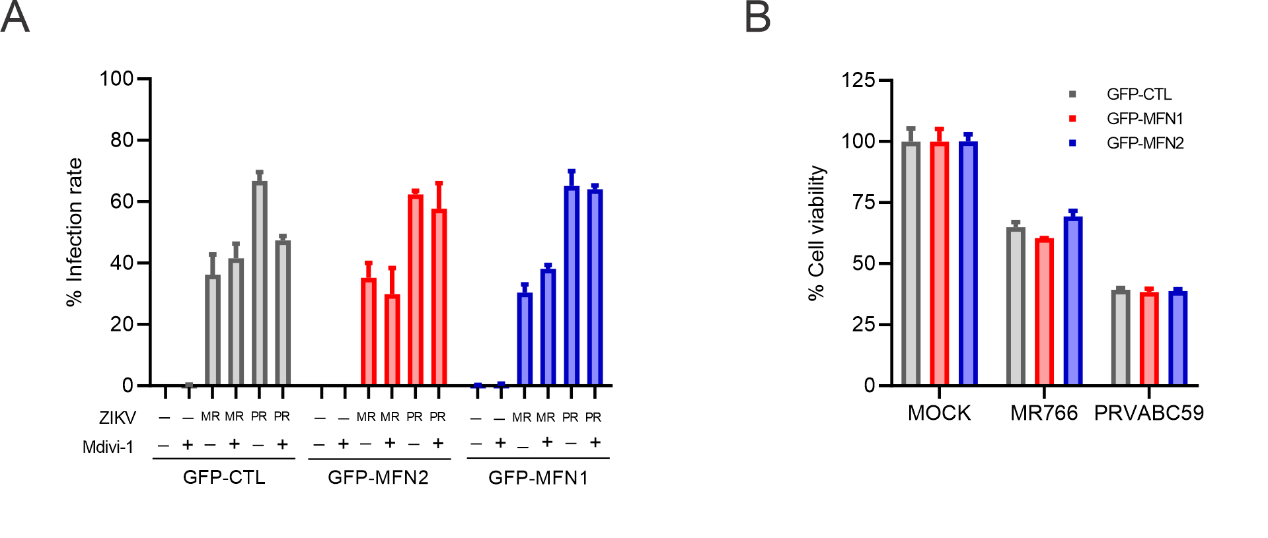


**Supplementary Figure S5. ZIKV NS4A participates in ZIKV-induced mitochondrial fragmentation.**

(**A**) The infection rate after 48 hours of infection with ZIKV at MOI=5 in the presence of 50 μM Mdivi-1 or DMSO. The values represent mean ± SD (n=3 replicates) (**B**) The viability of MFN over-expressing SNB-19 cells after 48 hours infection with ZIKV at MOI=5. The values represent mean ± SD (n=3 replicates)
